# Automating the Generation of Notifiable Bacterial Disease Reports: Proof-of-Concept Study and Implementation in Six Hospitals in Thailand

**DOI:** 10.4269/ajtmh.23-0848

**Published:** 2024-05-28

**Authors:** Cherry Lim, Preeyarach Klaytong, Viriya Hantrakun, Chalida Rangsiwutisak, Chadaporn Phiancharoen, Ratanaporn Tangwangvivat, Somkid Kripattanapong, Charuttaporn Jitpeera, Wiratya Poldech, Punya Jiramahasan, Bangon Laosatiankit, Orawan Photivet, Punchawee Sukbut, Warintorn Thongsri, Kailas Kosasaeng, Bongkoch Chiwehanyon, Nutjamee Leesahud, Preecha Ritthong, Wandee Linreung, Panatda Aramrueang, Wichan Bhunyakitikorn, Sopon Iamsirithaworn, Direk Limmathurotsakul

**Affiliations:** ^1^ Mahidol-Oxford Tropical Medicine Research Unit, Mahidol University, Bangkok, Thailand;; ^2^ Centre for Tropical Medicine and Global Health, Nuffield Department of Medicine, University of Oxford, Oxford, United Kingdom;; ^3^ Division of Communicable Diseases, Department of Disease Control, Ministry of Public Health, Nonthaburi, Thailand;; ^4^ Division of Epidemiology, Department of Disease Control, Ministry of Public Health, Nonthaburi, Thailand;; ^5^ Amnatcharoen Hospital, Amnatcharoen, Thailand;; ^6^ Kantharalak Hospital, Srisaket, Thailand;; ^7^ Srisaket Provincial Health Office, Srisaket, Thailand;; ^8^ Mukdahan Hospital, Mukdahan, Thailand;; ^9^ Nakhon Phanom Hospital, Nakhon Phanom, Thailand;; ^10^ Phatthalung Hospital, Phattalung, Thailand;; ^11^ Sunpasitthiprasong Hospital, Ubon Ratchathani, Thailand;; ^12^ Department of Tropical Hygiene, Faculty of Tropical Medicine, Mahidol University, Bangkok, Thailand

## Abstract

Information on notifiable bacterial diseases (NBD) in low- and middle-income countries (LMICs) is frequently incomplete. We developed the AutoMated tool for the Antimicrobial resistance Surveillance System plus (AMASSplus), which can support hospitals to analyze their microbiology and hospital data files automatically (in CSV or Excel format) and promptly generate antimicrobial resistance surveillance and NBD reports (in PDF and CSV formats). The NBD reports included the total number of cases and deaths after *Brucella* spp., *Burkholderia pseudomallei*, *Corynebacterium diphtheriae*, *Neisseria gonorrhoeae*, *Neisseria meningitidis*, nontyphoidal *Salmonella* spp., *Salmonella* enterica serovar Paratyphi, *Salmonella* enterica serovar Typhi, *Shigella* spp., *Streptococcus suis*, and *Vibrio* spp. infections. We tested the tool in six hospitals in Thailand in 2022. The total number of deaths identified by the AMASSplus was higher than those reported to the national notifiable disease surveillance system (NNDSS); particularly for *B. pseudomallei* infection (134 versus 2 deaths). This tool could support the NNDSS in LMICs.

The National Notifiable Disease Surveillance System (NNDSS) is established in most countries to control infections, detect outbreaks, and monitor the impact of interventions. The list of notifiable diseases may include a wide range of communicable and noncommunicable diseases such as anthrax, botulism, campylobacteriosis, and silicosis.^1^^,^^2^ The reporting methods vary across countries; some diseases require urgent notification within a few hours by phone, whereas others may be reported to defined systems within a certain time frame. For example, the Department of Disease control (DDC), Thailand, has the Director Critical Information Requirement (DCIR) criteria, which define the situations of notifiable diseases that must be notified within 2 hours.^3^ Many high-income countries have modernized the NNDSS and use automated and computerized data management system.^4^^,^^5^

The information of notifiable diseases reported to the NNDSS in low and middle-income countries (LMICs) is often incomplete. Most countries rely on manual (or semiautomated) reporting of cases by responsible officers in healthcare facilities.^6^ However, healthcare facilities frequently lack laboratory capacity to detect the notifiable diseases. Referral hospitals that can provide a definite diagnosis of a notifiable disease also lack the human resources to prepare, verify, and submit the data to the NNDSS. Priority diseases, such as COVID-19, could also reduce the data completeness of other notifiable diseases due to high levels of burnout.^7^

The incomplete information of notifiable diseases can have a negative impact on policymakers and stakeholders. For example, melioidosis,^8^ an infection cause by the Gram-negative bacteria *Burkholderia pseudomallei*, has been a notifiable disease in Thailand since 2002.^9^ The disease is highly prevalent and fatal in Thailand.^10^ However, only approximately 10 deaths from melioidosis are reported yearly to the NNDSS. This is mainly because referral hospitals do not report the cases and deaths of culture-confirmed melioidosis to the NNDSS.^9^^,^^10^ The Ministry of Public Health (MoPH) of Thailand advises against using the number of cases and deaths reported to the NNDSS to represent the disease burden. Nonetheless, policymakers and stakeholders often use those numbers to represent the burden of melioidosis in the country, which hinders the efforts to improve awareness, diagnosis, treatment, and prevention of melioidosis in Thailand.^9^

We recently developed the AutoMated tool for Antimicrobial resistance Surveillance System (AMASS), an offline application that can automatically analyze and generate standardized antimicrobial resistance (AMR) surveillance reports from routine microbiology and hospital data.^11^ We tested the application in seven hospitals in seven countries.^11^ The AMASS version 1.0 (called AMASSv1.0) was released on February 1, 2019.^11^ The aim of this study was to extend the AMASS to additionally analyze and generate notifiable bacterial disease (NBD) reports (called AMASSplus). The AMASSplus was released on March 25, 2021. In this study, we tested the AMASSplus in six referral hospitals with microbiology laboratories in Thailand. Among the six referral hospitals, only Sunpasitthiprasong Hospital participated in testing both AMASSv1.0^11^ and AMASSplus (in this study) but using data from different years.

We based the AMASSplus on the AMASSv1.0,^11^ which is an open access, user-friendly, and highly compatible application with high data security. The AMASSplus was similarly built and included both R portable (version 3.4.3; R Project for Statistical Computing) and RStudio (version 1.1.423; RStudio, Inc., Vienna, Austria) within the downloadable package so that the application can used without the need to install R or any program before running the application. The AMASSplus also uses data dictionary files (in Excel format) to accommodate data (in either CSV or Excel) exported from different systems or programs used by microbiology laboratories and hospitals that may have different ways to name data variables and data values. The AMASSplus can also be run by double clicking on the application file without any further user input. In addition to AMASSv1.0,^11^ the AMASSplus additionally generated the Annex reporting NBDs caused by 11 pathogens including *Brucella* spp., *Burkholderia pseudomallei*, *Corynebacterium diphtheriae*, *Neisseria gonorrhoeae*, *Neisseria meningitidis*, nontyphoidal *Salmonella* spp., *Salmonella enterica* serovar Paratyphi, *Salmonella* enterica serovar Typhi, *Shigella* spp., *Streptococcus suis*, and *Vibrio* spp. We selected the NBDs based on their priority in Thailand and in collaboration with the DDC, MoPH, Thailand. The NBD cases were defined as having a clinical specimen culture positive for a pathogen. The AMASSplus deduplicated the laboratory data by reporting the total number of patients with a clinical specimen culture positive for a pathogen during the evaluation period, using the hospital number (i.e., the patient identifier) and specimen collection dates. For each clinical specimen type (e.g., blood, cerebrospinal fluid, urine), the AMASSplus also deduplicated and reported the total number of patients with each clinical specimen type culture positive for a pathogen during the evaluation period. Then AMASSplus merged the deduplicated laboratory data with the hospital admission data, using the hospital number (i.e., the patient identifier) present in both data files.^11^ Mortality was defined using the discharge summary (in the hospital admission data), which was regularly completed by the attending physicians and reported to the MoPH. If a patient was admitted with an NBD more than once during the evaluation period, the mortality outcome of the first admission was presented.

In 2022, we tested the AMASSplus in six referral hospitals in six provinces, including Amnatcharoen Hospital, Kantharalak Hospital, Mukdahan Hospital, Nakhon Phanom Hospital, Phatthalung Hospital, and Sunpasitthiprasong Hospital (Figure 1). The hospitals were selected based on agreement with the DDC. We requested that each hospital independently export their hospital admission file (from their hospital information systems) and microbiology data file (from their laboratory information systems) as either CSV or Excel format and ran the AMASSplus. We held at least one virtual or face-to-face meeting with each hospital and additional online support as needed. Common issues observed were inaccurate formatting of date variables in the Excel files and incomplete data dictionary files, resulting in inaccurate reports.^11^ Together, the study team and the participating hospitals validated the reports and log files carefully.^11^ The participating hospitals then addressed the issues (e.g., correcting date formats and completing the data dictionary files), and reran the AMASSplus until the generated reports and log files confirmed their completeness. Ethical permission for this study was obtained from the Institute for the Committee of the Faculty of Tropical Medicine, Mahidol University (TMEC 23-086).

**Figure 1.**
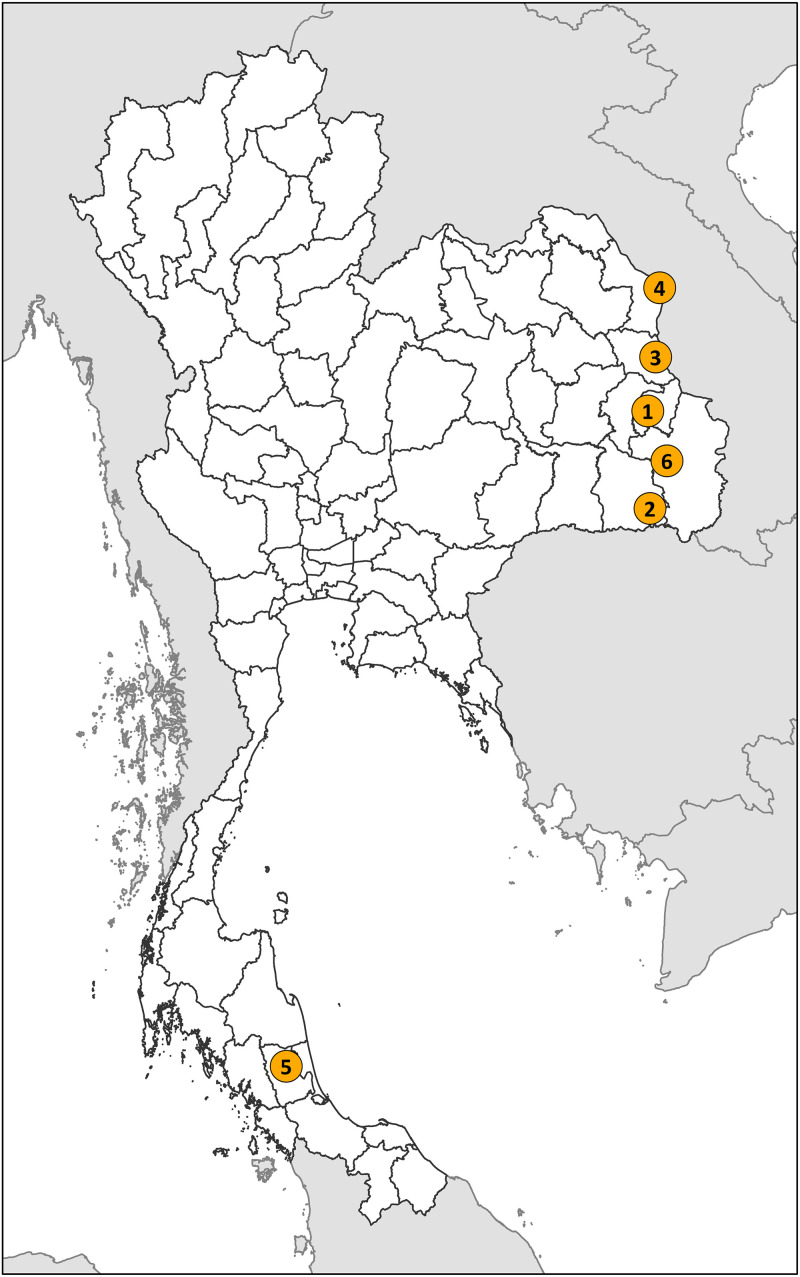
Locations of the six participating hospitals, including (1) Amnatcharoen Hospital, (2) Kantharalak Hospital, (3) Mukdahan Hospital, (4) Nakhon Phanom Hospital, (5) Phatthalung Hospital, and (6) Sunpasitthiprasong Hospital.

All six hospitals successfully generated and deposited their AMR plus NBD summary reports in an open-access platform.^12^^–^^17^ The median bed number was 453 (range: 281–1,158). Mukdahan Hospital and Sunpasitthiprasong Hospital used data from 2020, whereas the other four hospitals used the data from 2021. Sunpasitthiprasong Hospital changed its laboratory information system in 2020 and thus generated two reports. All hospitals included in this study obtained both microbiology data and hospital admission data (which included discharge summaries), except Amnatcharoen Hospital, which obtained only microbiology data. Therefore, mortality data from Amnatcharoen Hospital were not available.

During the evaluation period (Table 1), the NBD with the highest number of cases was *B. pseudomallei* infection (*n =* 834 patients), followed by infections with nontyphoidal *Salmonella* spp. (*n =* 281), *Vibrio* spp. (*n =* 78), *Streptococcus suis* (*n =* 32), *Neisseria gonorrhoeae* (*n =* 7), *Shigella* spp. (*n =* 3), *Brucella* spp. (*n =* 2), and *Corynebacterium diphtheriae* (*n =* 1). The NBD with the highest number of deaths was *B. pseudomallei* infection (*n =* 134 deaths), followed by nontyphoidal *Salmonella* spp. (*n =* 17), *Vibrio* spp. (*n =* 5), *S. suis* (*n =* 4), and *Shigella* spp. (*n =* 1).

**Table 1:** Total number of cases and deaths after notifiable bacterial diseases in six hospitals in Thailand

Notifiable Bacterial Diseases	Amnatcharoen Hospital	Kantharalak Hospital	Mukdahan Hospital	Nakhon Phanom Hospital	Phatthalung Hospital	Sunpasitthiprasong Hospital
Total no. of cases*						
*Brucella* spp.	0	0	1	0	0	1
*Burkholderia pseudomallei*	134	22	265	82	14	317
*Corynebacterium diphtheriae*	0	0	0	0	0	1
*Neisseria gonorrhoeae*	4	0	0	0	0	3
*Neisseria meningitidis*	0	0	0	0	0	0
Nontyphoidal *Salmonella* spp.	34	14	85	7	74	67
*Salmonella* enterica serovar Paratyphi	0	0	0	0	0	0
*Salmonella* enterica serovar Typhi	0	0	0	0	0	0
*Shigella* spp.	2	0	0	0	1	0
*Streptococcus suis*	0	4	0	5	3	20
*Vibrio* spp.	9	0	45	2	5	17
Mortality (%)^†^						
*Brucella* spp.	NA	N/A	0% (0/1)	N/A	N/A	0% (0/1)
*Burkholderia pseudomallei*	NA	5% (1/21)	14% (25/174)	32% (23/73)	18% (2/11)	29% (83/283)
*Corynebacterium diphtheriae*	NA	N/A	N/A	N/A	N/A	0% (0/1)
*Neisseria gonorrhoeae*	NA	N/A	N/A	N/A	N/A	0% (0/1)
*Neisseria meningitidis*	NA	N/A	N/A	N/A	N/A	N/A
Nontyphoidal *Salmonella* spp.	NA	8% (1/12)	6% (4/68)	20% (1/5)	11% (4/37)	12% (7/57)
*Salmonella* enterica serovar Paratyphi	NA	N/A	N/A	N/A	N/A	N/A
*Salmonella* enterica serovar Typhi	NA	N/A	N/A	N/A	N/A	N/A
*Shigella* spp.	NA	N/A	N/A	N/A	100% (1/1)	N/A
*Streptococcus suis*	NA	0% (0/3)	N/A	0% (0/4)	100% (1/1)	2% (3/18)
*Vibrio* spp.	NA	N/A	2% (1/40)	50% (1/2)	100% (1/1)	1% (2/14)

NA = not available; N/A = not applicable.

* AutoMated tool for the Antimicrobial resistance Surveillance System plus (AMASSplus) identified notifiable bacterial diseases cases with a clinical specimen culture positive for a pathogen during the evaluation period. Total number of cases identified by the AMASSplus included outpatient cases, inpatient cases and cases whose only clinical specimens were sent to the microbiology laboratories of the study hospitals.

^†^ Mortality was estimated among inpatient cases. Death was defined using the discharge summary regularly completed by the attending physicians and reported to the Ministry of Public Health. Amnatcharoen Hospital did not obtain hospital admission data file and used only microbiology data file. Therefore, mortality data was not available.

We compared total number of cases and deaths following culture-confirmed NBDs identified by the AMASSplus with the relevant NBDs reported to the NNDSS from the six provinces during the same period (Table 2).^18^ For melioidosis, gonorrhea, food poisoning, typhoid, and shigellosis, the total number of cases identified by the AMASSplus were lower than those reported to the NNDSS. This was because the NNDSS included reports of suspected, probable, and confirmed cases using a wide range of case definitions of each notifiable disease,^19^ whereas the AMASSplus only identified patients with culture positivity. The total number of cases with *Brucella* spp., *C. diptheriae*, and *S. suis* infections identified by the AMASSplus were higher than those reported to the NNDSS. For NBDs with fatality cases, the total number of deaths identified by the AMASSplus was higher than those reported to the NNDSS; particularly for *B. pseudomallei* infection (134 versus two deaths, Table 2).

**Table 2:** Total number of cases and deaths after culture-confirmed NBDs identified using the AMASSplus in six participating hospitals compared with total number of cases and deaths with relevant notifiable diseases reported to the NNDSS in six provinces during the same period

Notifiable Bacterial Diseases	AMASSplus	Relevant Notifiable Diseases in the NNDSS	NNDSS
Total number of cases*			
*Brucella* spp.	2	Brucellosis	1
*Burkholderia pseudomallei*	834	Melioidosis	953
*Corynebacterium diphtheriae*	1	Diphtheria	0
*Neisseria gonorrhoeae*	7	Gonorrhea	1,009
*Neisseria meningitidis*	0	Meningococcal meningitis	0
Nontyphoidal *Salmonella* spp. and *Vibrio* spp.	359	Food poisoning and cholera^†^	12,659
*Salmonella* enterica serovar Paratyphi and Typhi	0	Typhoid and paratyphoid^‡^	11
*Shigella* spp.	3	Shigellosis	63
*Streptococcus suis*	32	*Streptococcus suis* infection	5
Total number of deaths^§^			
*Brucella* spp.	0	Brucellosis	0
*Burkholderia pseudomallei*	134	Melioidosis	2
*Corynebacterium diphtheriae*	0	Diphtheria	0
*Neisseria gonorrhoeae*	0	Gonorrhea	0
*Neisseria meningitidis*	0	Meningococcal meningitis	0
Nontyphoidal *Salmonella* spp. and *Vibrio* spp.	22	Food poisoning and cholera^‖^	1
*Salmonella* enterica serovar Paratyphi and Typhi	0	Typhoid and paratyphoid	0
*Shigella* spp.	1	Shigellosis	0
*Streptococcus suis*	4	*Streptococcus suis*	2

AMASSplus = AutoMated tool for the Antimicrobial resistance Surveillance System plus; NBDs = notifiable bacterial diseases; NNDSS = national notifiable disease surveillance systems.

* The AMASSplus identified culture-confirmed NBDs with a clinical specimen culture positive for a pathogen during the evaluation period, whereas the NNDSS included reports of suspected, probable, and confirmed cases using a wide range of case definitions of each notifiable disease.^18^^,^^19^

^†^ Cases with food poisoning (*n =* 12,659) and cholera (*n =* 0).

^‡^ Case with typhoid (*n =* 11) and paratyphoid (*n =* 0).

^§^ Total number of deaths after relevant notifiable diseases in the NNDSS were from five provinces: Mukdahan, Nakhon Phanom, Phattalung, Srisaket, and Ubon Ratchathani (excluding Amnatcharoen province).

^‖^ Deaths following food poisoning (*n =* 1) and cholera (*n =* 0).

This study demonstrated the feasibility of using the AMASSplus to report the incidence and mortality of culture-confirmed NBDs in LMICs. We showed that electronic data of microbiology laboratories and hospital admission records could be used to support the information of NBDs to the NNDSS. The high burden of melioidosis is consistent with the previous clinical studies.^9^^,^^10^ This user-friendly and time-efficient tool enables individual hospitals to generate and share standardized reports in a timely manner. This could facilitate collaborative works across different settings, both nationally and globally, to support prioritization of public health resources.

We discussed the findings with the staff in each participating hospital and shared the reports with the DDC of the MoPH. The main barriers for the referral hospitals to report NBDs were that all levels of healthcare workers are not aware that those culture-confirmed cases should be reported to the NNDSS and that the responsible officers use the *International Classification of Diseases*, 10th revision (ICD-10) without using culture results to identify and report NBDs. However, the ICD-10 codings are often delayed, incomplete, or inaccurate.

The AMASSplus is now considered as an important tool by the DDC, MoPH of Thailand. The key measure of the national plan for melioidosis prevention and control, launched by the DDC in 2021, is to improve melioidosis surveillance.^20^ The DDC is using the AMASSplus to understand the total number of cases and deaths after culture-confirmed NBDs nationwide and supplement the information of NBDs officially reported to the NNDSS. The additional information of culture-confirmed NBDs would help the DDC design strategic interventions and allocate resources against the NBDs.

The study has some limitations. Comparisons of incidence and mortality between hospitals and between diseases need to be made with great caution because there are multiple confounding factors. The reported mortality could be an underestimation because the tool cannot identify moribund patients who were discharged against advice and died at home. Moreover, the reported mortality was based on the first admission, which had a clinical specimen culture positive for a pathogen and could be an underestimation for patients who experienced multiple readmissions due to the NBDs. The reports generated by the AMASSplus are designed to be retrospective and could not be used in outbreak situations as an alert system.

In conclusion, the AMASSplus can support the information of NBDs in Thailand. We suggest that stakeholders in LMICs should consider supporting hospitals that have microbiology laboratories and electronic data records to use any appropriate analytical software or tools, analyze and generate reports on NBDs, and use the data for their actions and decision-making.

## References

[1] Thomas K Jajosky R Coates RJ Calvert GM Dewey-Mattia D Raymond J Singh SD, 2017. Summary of notifiable noninfectious conditions and disease outbreaks: Surveillance data published between April 1, 2016 and January 31, 2017 – United States. MMWR Morb Mortal Wkly Rep 64: 1–6. 10.15585/mmwr.mm6454a1 28796765

[2] Adams DA Thomas KR Jajosky RA Foster L Baroi G Sharp P Onweh DH Schley AW Anderson WJ, for the Nationally Notifiable Infectious Conditions Group , 2017. Summary of notifiable infectious diseases and conditions – United States, 2015. MMWR Morb Mortal Wkly Rep 64: 1–143. 10.15585/mmwr.mm6453a1 28796757

[3] Division of Epidemiology, Department of Disease Control, Ministry of Public Health, Thailand , 2020. *Standard Operating Procedures for Surveillance and Rapid Response Team, Thailand, 2020*. Available at: https://ddc.moph.go.th/uploads/publish/1119320210312043053.pdf. Accessed November 11, 2022.

[4] Azar FE Masoori N Meidani Z Paul L, 2010. Proposal for a modernized Iranian notifiable infectious diseases surveillance system: Comparison with USA and Australia. East Mediterr Health J 16: 771–777. 20799535

[5] Vlieg WL , 2017. Comparing national infectious disease surveillance systems: China and the Netherlands. BMC Public Health 17: 415. 28482830 10.1186/s12889-017-4319-3 PMC5423001

[6] Jayatilleke K, 2020. Challenges in implementing surveillance tools of high-income countries (HICs) in low middle income countries (LMICs). Curr Treat Options Infect Dis 12: 191–201. 32874140 10.1007/s40506-020-00229-2 PMC7453076

[7] Nansikombi HT Kwesiga B Aceng FL Ario AR Bulage L Arinaitwe ES, 2023. Timeliness and completeness of weekly surveillance data reporting on epidemic prone diseases in Uganda, 2020–2021. BMC Public Health 23: 647. 37016380 10.1186/s12889-023-15534-w PMC10072024

[8] Meumann EM Limmathurotsakul D Dunachie SJ Wiersinga WJ Currie BJ, 2023. *Burkholderia pseudomallei* and melioidosis. Nat Rev Microbiol 22: 155–169. 37794173 10.1038/s41579-023-00972-5

[9] Hinjoy S , 2018. Melioidosis in Thailand: Present and future. Trop Med Infect Dis 3: 38. 29725623 10.3390/tropicalmed3020038 PMC5928800

[10] Hantrakun V Kongyu S Klaytong P Rongsumlee S Day NPJ Peacock SJ Hinjoy S Limmathurotsakul D, 2019. Clinical epidemiology of 7126 melioidosis patients in Thailand and the Implications for a National Notifiable Diseases Surveillance System. Open Forum Infect Dis 6: ofz498. 32083145 10.1093/ofid/ofz498 PMC7020769

[11] Lim C , 2020. Automating the generation of antimicrobial resistance surveillance reports: Proof-of-concept study involving seven hospitals in seven countries. J Med Internet Res 22: e19762. 33006570 10.2196/19762 PMC7568216

[12] Amnatcharoen Hospital , 2022. *Antimicrobial Resistance Surveillance Report, Amnatcharoen Hospital, Amnatcharoen, Thailand, 01 Jan 2021 to 31 Dec 2021*. Available at: 10.6084/m9.figshare.19709929. Accessed November 11, 2022.

[13] Kantharalak Hospital , 2022. *Antimicrobial Resistance Surveillance Report, Kantharalak Hospital, Sisaket, Thailand, 01 Jan 2021 to 31 Dec 2021*. Available at: 10.6084/m9.figshare.19706662. Accessed November 11, 2022.

[14] Mukdahan, Thailand , 2022. *Antimicrobial Resistance Surveillance Report, Mukdahan Hospital, Mukdahan, Thailand, 01 Jan 2020 to 31 Jan 2021*. Available at: 10.6084/m9.figshare.19710568. Accessed November 11, 2022.

[15] Nakhonphanom Hospital , 2022. *Antimicrobial Resistance Surveillance Report, Nakhonphanom Hospital, Nakhonphanom, Thailand, 01 Jan 2021 to 31 Dec 2021*. Available at: 10.6084/m9.figshare.19709911. Accessed November 11, 2022.

[16] Phatthalung Hospital , 2022. *Antimicrobial Resistance Surveillance Report, Phatthalung Hospital, Phatthalung, Thailand, 01 Jan 2021 to 26 Aug 2021*. Available at: 10.6084/m9.figshare.19706728. Accessed November 11, 2022.

[17] Sunpasitthiprasong Hospital , 2022. *Antimicrobial Resistance Surveillance Report, Sunpasitthiprasong Hospital, Ubon Ratchathani, Thailand, 23 Jul 2020 to 31 Jan 2021*. Available at: 10.6084/m9.figshare.19710724. Accessed November 11, 2022.

[18] Division of Epidemiology, Department of Disease Control Ministry of Public Health, Thailand *National Disease Surveillance (Report 506).* Available at: http://doe.moph.go.th/surdata/. Accessed November 11, 2022.

[19] Division of Epidemiology , *Department of Disease Control, Ministry of Public Health, Thailand, 2020. Case definition for Communicable Diseases Surveillance, Thailand, 2020*. Available at: https://ddc.moph.go.th/uploads/publish/1142920210518092542.pdf. Accessed November 11, 2022.

[20] Bureau of General Communicable Diseases, Department of Disease Control, Ministry of Public Health, Thailand , 2021. *Melioidosis Guideline*. Available at: http://klb.ddc.moph.go.th/dataentry/handbook/form/129. Accessed November 11, 2022.

